# Circulating short-chain and branched short-chain fatty acids and the risk of incident type 2 diabetes: findings from the 4C study

**DOI:** 10.1093/lifemeta/loaf001

**Published:** 2025-01-22

**Authors:** Shuangyuan Wang, Hong Lin, Xiaojing Jia, Yiting Lin, Chunyan Hu, Mian Li, Yu Xu, Min Xu, Jie Zheng, Xinjie Zhao, Yanli Li, Lulu Chen, Tianshu Zeng, Ruying Hu, Zhen Ye, Lixin Shi, Qing Su, Yuhong Chen, Xuefeng Yu, Li Yan, Tiange Wang, Zhiyun Zhao, Guijun Qin, Qin Wan, Gang Chen, Meng Dai, Di Zhang, Bihan Qiu, Xiaoyan Zhu, Ruixin Liu, Xiao Wang, Xulei Tang, Zhengnan Gao, Feixia Shen, Xuejiang Gu, Zuojie Luo, Yingfen Qin, Li Chen, Xinguo Hou, Yanan Huo, Qiang Li, Guixia Wang, Yinfei Zhang, Chao Liu, Youmin Wang, Shengli Wu, Tao Yang, Huacong Deng, Jiajun Zhao, Yiming Mu, Guowang Xu, Shenghan Lai, Donghui Li, Guang Ning, Weiqing Wang, Yufang Bi, Jieli Lu

**Affiliations:** Department of Endocrine and Metabolic Diseases, Shanghai Institute of Endocrine and Metabolic Diseases, Ruijin Hospital, Shanghai Jiao Tong University School of Medicine, Shanghai 200025, China; Shanghai National Clinical Research Center for Endocrine and Metabolic Diseases, Key Laboratory for Endocrine and Metabolic Diseases of the National Health Commission of the People’s Republic of China, Shanghai National Center for Translational Medicine, Ruijin Hospital, Shanghai Jiao Tong University School of Medicine, Shanghai 200025, China; Department of Endocrine and Metabolic Diseases, Shanghai Institute of Endocrine and Metabolic Diseases, Ruijin Hospital, Shanghai Jiao Tong University School of Medicine, Shanghai 200025, China; Shanghai National Clinical Research Center for Endocrine and Metabolic Diseases, Key Laboratory for Endocrine and Metabolic Diseases of the National Health Commission of the People’s Republic of China, Shanghai National Center for Translational Medicine, Ruijin Hospital, Shanghai Jiao Tong University School of Medicine, Shanghai 200025, China; Department of Endocrine and Metabolic Diseases, Shanghai Institute of Endocrine and Metabolic Diseases, Ruijin Hospital, Shanghai Jiao Tong University School of Medicine, Shanghai 200025, China; Shanghai National Clinical Research Center for Endocrine and Metabolic Diseases, Key Laboratory for Endocrine and Metabolic Diseases of the National Health Commission of the People’s Republic of China, Shanghai National Center for Translational Medicine, Ruijin Hospital, Shanghai Jiao Tong University School of Medicine, Shanghai 200025, China; Department of Endocrine and Metabolic Diseases, Shanghai Institute of Endocrine and Metabolic Diseases, Ruijin Hospital, Shanghai Jiao Tong University School of Medicine, Shanghai 200025, China; Shanghai National Clinical Research Center for Endocrine and Metabolic Diseases, Key Laboratory for Endocrine and Metabolic Diseases of the National Health Commission of the People’s Republic of China, Shanghai National Center for Translational Medicine, Ruijin Hospital, Shanghai Jiao Tong University School of Medicine, Shanghai 200025, China; Department of Endocrine and Metabolic Diseases, Shanghai Institute of Endocrine and Metabolic Diseases, Ruijin Hospital, Shanghai Jiao Tong University School of Medicine, Shanghai 200025, China; Shanghai National Clinical Research Center for Endocrine and Metabolic Diseases, Key Laboratory for Endocrine and Metabolic Diseases of the National Health Commission of the People’s Republic of China, Shanghai National Center for Translational Medicine, Ruijin Hospital, Shanghai Jiao Tong University School of Medicine, Shanghai 200025, China; Department of Endocrine and Metabolic Diseases, Shanghai Institute of Endocrine and Metabolic Diseases, Ruijin Hospital, Shanghai Jiao Tong University School of Medicine, Shanghai 200025, China; Shanghai National Clinical Research Center for Endocrine and Metabolic Diseases, Key Laboratory for Endocrine and Metabolic Diseases of the National Health Commission of the People’s Republic of China, Shanghai National Center for Translational Medicine, Ruijin Hospital, Shanghai Jiao Tong University School of Medicine, Shanghai 200025, China; Department of Endocrine and Metabolic Diseases, Shanghai Institute of Endocrine and Metabolic Diseases, Ruijin Hospital, Shanghai Jiao Tong University School of Medicine, Shanghai 200025, China; Shanghai National Clinical Research Center for Endocrine and Metabolic Diseases, Key Laboratory for Endocrine and Metabolic Diseases of the National Health Commission of the People’s Republic of China, Shanghai National Center for Translational Medicine, Ruijin Hospital, Shanghai Jiao Tong University School of Medicine, Shanghai 200025, China; Department of Endocrine and Metabolic Diseases, Shanghai Institute of Endocrine and Metabolic Diseases, Ruijin Hospital, Shanghai Jiao Tong University School of Medicine, Shanghai 200025, China; Shanghai National Clinical Research Center for Endocrine and Metabolic Diseases, Key Laboratory for Endocrine and Metabolic Diseases of the National Health Commission of the People’s Republic of China, Shanghai National Center for Translational Medicine, Ruijin Hospital, Shanghai Jiao Tong University School of Medicine, Shanghai 200025, China; Department of Endocrine and Metabolic Diseases, Shanghai Institute of Endocrine and Metabolic Diseases, Ruijin Hospital, Shanghai Jiao Tong University School of Medicine, Shanghai 200025, China; Shanghai National Clinical Research Center for Endocrine and Metabolic Diseases, Key Laboratory for Endocrine and Metabolic Diseases of the National Health Commission of the People’s Republic of China, Shanghai National Center for Translational Medicine, Ruijin Hospital, Shanghai Jiao Tong University School of Medicine, Shanghai 200025, China; Key Laboratory of Separation Science for Analytical Chemistry, Dalian Institute of Chemical Physics, Chinese Academy of Sciences, Dalian, Liaoning 116023, China; Key Laboratory of Separation Science for Analytical Chemistry, Dalian Institute of Chemical Physics, Chinese Academy of Sciences, Dalian, Liaoning 116023, China; Department of Endocrine and Metabolic Diseases, Union Hospital, Tongji Medical College, Huazhong University of Science and Technology, Wuhan, Hubei 430022, China; Department of Endocrine and Metabolic Diseases, Union Hospital, Tongji Medical College, Huazhong University of Science and Technology, Wuhan, Hubei 430022, China; Zhejiang Provincial Center for Disease Control and Prevention, Hangzhou, Zhejiang 310051, China; Zhejiang Provincial Center for Disease Control and Prevention, Hangzhou, Zhejiang 310051, China; Department of Endocrine and Metabolic Diseases, Affiliated Hospital of Guiyang Medical College, Guiyang, Guizhou 550004, China; Department of Endocrine and Metabolic Diseases, Xinhua Hospital Affiliated to Shanghai Jiao Tong University School of Medicine, Shanghai 200092, China; Department of Endocrine and Metabolic Diseases, Shanghai Institute of Endocrine and Metabolic Diseases, Ruijin Hospital, Shanghai Jiao Tong University School of Medicine, Shanghai 200025, China; Shanghai National Clinical Research Center for Endocrine and Metabolic Diseases, Key Laboratory for Endocrine and Metabolic Diseases of the National Health Commission of the People’s Republic of China, Shanghai National Center for Translational Medicine, Ruijin Hospital, Shanghai Jiao Tong University School of Medicine, Shanghai 200025, China; Department of Endocrine and Metabolic Diseases, Tongji Hospital, Tongji Medical College, Huazhong University of Science and Technology, Wuhan, Hubei 430030, China; Department of Endocrine and Metabolic Diseases, Sun Yat-sen Memorial Hospital, Sun Yat-sen University, Guangzhou, Guangdong 510120, China; Department of Endocrine and Metabolic Diseases, Shanghai Institute of Endocrine and Metabolic Diseases, Ruijin Hospital, Shanghai Jiao Tong University School of Medicine, Shanghai 200025, China; Shanghai National Clinical Research Center for Endocrine and Metabolic Diseases, Key Laboratory for Endocrine and Metabolic Diseases of the National Health Commission of the People’s Republic of China, Shanghai National Center for Translational Medicine, Ruijin Hospital, Shanghai Jiao Tong University School of Medicine, Shanghai 200025, China; Department of Endocrine and Metabolic Diseases, Shanghai Institute of Endocrine and Metabolic Diseases, Ruijin Hospital, Shanghai Jiao Tong University School of Medicine, Shanghai 200025, China; Shanghai National Clinical Research Center for Endocrine and Metabolic Diseases, Key Laboratory for Endocrine and Metabolic Diseases of the National Health Commission of the People’s Republic of China, Shanghai National Center for Translational Medicine, Ruijin Hospital, Shanghai Jiao Tong University School of Medicine, Shanghai 200025, China; Department of Endocrine and Metabolic Diseases, The First Affiliated Hospital of Zhengzhou University, Zhengzhou, Henan 450052, China; Department of Endocrine and Metabolic Diseases, The Affiliated Hospital of Southwest Medical University, Luzhou, Sichuan 646000, China; Department of Endocrine and Metabolic Diseases, Fujian Provincial Hospital, Fujian Medical University, Fuzhou, Fujian 350003, China; Department of Endocrine and Metabolic Diseases, Shanghai Institute of Endocrine and Metabolic Diseases, Ruijin Hospital, Shanghai Jiao Tong University School of Medicine, Shanghai 200025, China; Shanghai National Clinical Research Center for Endocrine and Metabolic Diseases, Key Laboratory for Endocrine and Metabolic Diseases of the National Health Commission of the People’s Republic of China, Shanghai National Center for Translational Medicine, Ruijin Hospital, Shanghai Jiao Tong University School of Medicine, Shanghai 200025, China; Department of Endocrine and Metabolic Diseases, Shanghai Institute of Endocrine and Metabolic Diseases, Ruijin Hospital, Shanghai Jiao Tong University School of Medicine, Shanghai 200025, China; Shanghai National Clinical Research Center for Endocrine and Metabolic Diseases, Key Laboratory for Endocrine and Metabolic Diseases of the National Health Commission of the People’s Republic of China, Shanghai National Center for Translational Medicine, Ruijin Hospital, Shanghai Jiao Tong University School of Medicine, Shanghai 200025, China; Department of Endocrine and Metabolic Diseases, Shanghai Institute of Endocrine and Metabolic Diseases, Ruijin Hospital, Shanghai Jiao Tong University School of Medicine, Shanghai 200025, China; Shanghai National Clinical Research Center for Endocrine and Metabolic Diseases, Key Laboratory for Endocrine and Metabolic Diseases of the National Health Commission of the People’s Republic of China, Shanghai National Center for Translational Medicine, Ruijin Hospital, Shanghai Jiao Tong University School of Medicine, Shanghai 200025, China; Department of Endocrine and Metabolic Diseases, Shanghai Institute of Endocrine and Metabolic Diseases, Ruijin Hospital, Shanghai Jiao Tong University School of Medicine, Shanghai 200025, China; Shanghai National Clinical Research Center for Endocrine and Metabolic Diseases, Key Laboratory for Endocrine and Metabolic Diseases of the National Health Commission of the People’s Republic of China, Shanghai National Center for Translational Medicine, Ruijin Hospital, Shanghai Jiao Tong University School of Medicine, Shanghai 200025, China; Department of Endocrine and Metabolic Diseases, Shanghai Institute of Endocrine and Metabolic Diseases, Ruijin Hospital, Shanghai Jiao Tong University School of Medicine, Shanghai 200025, China; Shanghai National Clinical Research Center for Endocrine and Metabolic Diseases, Key Laboratory for Endocrine and Metabolic Diseases of the National Health Commission of the People’s Republic of China, Shanghai National Center for Translational Medicine, Ruijin Hospital, Shanghai Jiao Tong University School of Medicine, Shanghai 200025, China; Department of Endocrine and Metabolic Diseases, Shanghai Institute of Endocrine and Metabolic Diseases, Ruijin Hospital, Shanghai Jiao Tong University School of Medicine, Shanghai 200025, China; Shanghai National Clinical Research Center for Endocrine and Metabolic Diseases, Key Laboratory for Endocrine and Metabolic Diseases of the National Health Commission of the People’s Republic of China, Shanghai National Center for Translational Medicine, Ruijin Hospital, Shanghai Jiao Tong University School of Medicine, Shanghai 200025, China; Department of Endocrine and Metabolic Diseases, The First Hospital of Lanzhou University, Lanzhou, Gansu 730000, China; Department of Endocrine and Metabolic Diseases, Dalian Municipal Central Hospital, Dalian, Liaoning 116033, China; Department of Endocrine and Metabolic Diseases, The First Affiliated Hospital of Wenzhou Medical University, Wenzhou, Zhejiang 325000, China; Department of Endocrine and Metabolic Diseases, The First Affiliated Hospital of Wenzhou Medical University, Wenzhou, Zhejiang 325000, China; Department of Endocrine and Metabolic Diseases, The First Affiliated Hospital of Guangxi Medical University, Nanning, Guangxi 530021, China; Department of Endocrine and Metabolic Diseases, The First Affiliated Hospital of Guangxi Medical University, Nanning, Guangxi 530021, China; Department of Endocrine and Metabolic Diseases, Qilu Hospital of Shandong University, Jinan, Shandong 250012, China; Department of Endocrine and Metabolic Diseases, Qilu Hospital of Shandong University, Jinan, Shandong 250012, China; Department of Endocrine and Metabolic Diseases, Jiangxi Provincial People’s Hospital Affiliated to Nanchang University, Nanchang, Jiangxi 330006, China; Department of Endocrine and Metabolic Diseases, The Second Affiliated Hospital of Harbin Medical University, Harbin, Heilongjiang 150086, China; Department of Endocrine and Metabolic Diseases, The First Hospital of Jilin University, Changchun, Jilin 130021, China; Department of Endocrine and Metabolic Diseases, Central Hospital of Shanghai Jiading District, Shanghai 201800, China; Department of Endocrine and Metabolic Diseases, Jiangsu Province Hospital on Integration of Chinese and Western Medicine, Nanjing, Jiangsu 210028, China; Department of Endocrine and Metabolic Diseases, The First Affiliated Hospital of Anhui Medical University, Hefei, Anhui 230022, China; Department of Endocrine and Metabolic Diseases, Karamay Municipal People’s Hospital, Karamay, Xinjiang 834000, China; Department of Endocrine and Metabolic Diseases, The First Affiliated Hospital of Nanjing Medical University, Nanjing, Jiangsu 210029, China; Department of Endocrine and Metabolic Diseases, The First Affiliated Hospital of Chongqing Medical University, Chongqing 400016, China; Department of Endocrine and Metabolic Diseases, Shandong Provincial Hospital affiliated to Shandong University, Jinan, Shandong 250021, China; Department of Endocrine and Metabolic Diseases, Chinese People’s Liberation Army General Hospital, Beijing 100853, China; Key Laboratory of Separation Science for Analytical Chemistry, Dalian Institute of Chemical Physics, Chinese Academy of Sciences, Dalian, Liaoning 116023, China; Department of Epidemiology and Public Health, University of Maryland School of Medicine, Baltimore, MD 21201, United States; Department of Gastrointestinal Medical Oncology, the University of Texas MD Anderson Cancer Center, Houston, TX 77030, United States; Department of Endocrine and Metabolic Diseases, Shanghai Institute of Endocrine and Metabolic Diseases, Ruijin Hospital, Shanghai Jiao Tong University School of Medicine, Shanghai 200025, China; Shanghai National Clinical Research Center for Endocrine and Metabolic Diseases, Key Laboratory for Endocrine and Metabolic Diseases of the National Health Commission of the People’s Republic of China, Shanghai National Center for Translational Medicine, Ruijin Hospital, Shanghai Jiao Tong University School of Medicine, Shanghai 200025, China; Department of Endocrine and Metabolic Diseases, Shanghai Institute of Endocrine and Metabolic Diseases, Ruijin Hospital, Shanghai Jiao Tong University School of Medicine, Shanghai 200025, China; Shanghai National Clinical Research Center for Endocrine and Metabolic Diseases, Key Laboratory for Endocrine and Metabolic Diseases of the National Health Commission of the People’s Republic of China, Shanghai National Center for Translational Medicine, Ruijin Hospital, Shanghai Jiao Tong University School of Medicine, Shanghai 200025, China; Department of Endocrine and Metabolic Diseases, Shanghai Institute of Endocrine and Metabolic Diseases, Ruijin Hospital, Shanghai Jiao Tong University School of Medicine, Shanghai 200025, China; Shanghai National Clinical Research Center for Endocrine and Metabolic Diseases, Key Laboratory for Endocrine and Metabolic Diseases of the National Health Commission of the People’s Republic of China, Shanghai National Center for Translational Medicine, Ruijin Hospital, Shanghai Jiao Tong University School of Medicine, Shanghai 200025, China; Department of Endocrine and Metabolic Diseases, Shanghai Institute of Endocrine and Metabolic Diseases, Ruijin Hospital, Shanghai Jiao Tong University School of Medicine, Shanghai 200025, China; Shanghai National Clinical Research Center for Endocrine and Metabolic Diseases, Key Laboratory for Endocrine and Metabolic Diseases of the National Health Commission of the People’s Republic of China, Shanghai National Center for Translational Medicine, Ruijin Hospital, Shanghai Jiao Tong University School of Medicine, Shanghai 200025, China

**Keywords:** short-chain fatty acids, branched short-chain fatty acids, type 2 diabetes, insulin resistance

## Abstract

Previous studies suggested that fecal short-chain fatty acids (SCFAs) and branched short-chain fatty acids (BCFAs) are associated with glucose regulation. However, the potential relationship between circulating SCFAs and BCFAs with incident diabetes risk in both men and women remains unidentified in prospective cohort studies. In this study, we examined a panel of nine serum SCFAs and BCFAs in 3414 subjects with incident diabetes, and matched normoglycemic controls from the China Cardiometabolic Disease and Cancer Cohort study. In fully adjusted conditional logistic regression models, total SCFAs, total BCFAs, and isovaleric acid were significantly associated with incident type 2 diabetes mellitus (T2DM) (*P* < 0.05). Interestingly, gender-specific analysis showed that per standard deviation (SD) increment of SCFAs were positively associated with incident T2DM among women, with the odds ratio (95% confidence interval) of 1.16 (1.05–1.29) for total SCFAs and 1.18 (1.07–1.31) for propionate, respectively (*P* < 0.05, false discovery rate (FDR) < 0.05). No significant associations were observed in men. A significant interaction was detected between men and women for propionate (*P*_interaction_ < 0.001, FDR < 0.01). After further adjustment of insulin measurements, the associations of serum propionate with diabetes remained significant (*P* < 0.05, FDR < 0.05). Meanwhile, the associations of total BCFAs and isovaleric acid with diabetes were partially mediated by triglycerides, insulin resistance, and β-cell function in mediation analysis. These findings, for the first time in a large prospective cohort, provide evidence for an association between circulating SCFAs and BCFAs with T2DM risk, and support the potential role of circulating propionate with gender disparities in the early pathogenesis of diabetes.

## Introduction

Short-chain fatty acids (SCFAs) are defined as carboxylic acids with carbon atom numbers below six, and they serve as key signaling molecules linking gut microbiota and host health [1]. Acetate, propionate, butyrate, valeric acid, and hexanoic acid are the primary SCFAs mainly derived from the fermentation of indigestible carbohydrates. Microbially produced SCFAs in the colon are generally acknowledged for their beneficial effects on cardiometabolic health [2, 3], including enhancing insulin secretion, reducing plasma cholesterol and glucose levels, and controlling energy intake through the modulation of enteroendocrine hormones [4, 5]. In addition to the aforementioned SCFAs, isobutyric acid, isovaleric acid, 3-methylvaleric acid, and 4-methylvaleric acid, commonly referred to as branched short-chain fatty acids (BCFAs), are produced in the gut through the proteolytic fermentation of branched-chain amino acids (BCAAs) [6, 7]. Colonic BCFAs possess unique chemical structures contributing to their differential biological effects and have been linked to adverse health outcomes [8, 9]. More recently, BCFAs have been suggested to play a role in regulating adipocyte lipid and glucose metabolism [10], although the evidence on the effect of gut-derived BCFAs on type 2 diabetes mellitus (T2DM) remains limited.

Indeed, microbially produced SCFAs and BCFAs have been shown to reach the systemic circulation at micromolar concentrations, which is more directly linked to metabolic health parameters rather than its levels in the colon [11]. However, limited research has been conducted to assess the concentrations of serum SCFAs, and evidence exploring associations of different blood SCFAs with the risk of T2DM is scarce. Recently, a study of 43 patients with T2DM and 28 overweight/obese individuals indicated that circulating SCFAs, which are typically low in the T2DM group, are inversely associated with blood glucose and the homeostasis model assessment of insulin resistance (HOMA-IR) index [12]. Conversely, conflicting evidence from a cohort of 100 Chinese individuals aged 40–60 years suggested higher SCFA concentrations in patients with T2DM [13], raising doubts about whether SCFAs act as beneficial signaling molecules in the systemic circulation.

Previous studies on circulating SCFAs have yielded inconclusive results, primarily due to their small sample sizes and cross-sectional design. Additionally, these studies have mainly focused on comparing SCFA levels under different health statuses without analyzing their predictive role on incident T2DM risk. To address these gaps in knowledge, we conducted a nested case–control study using data from the nationwide, population-based, prospective China Cardiometabolic Disease and Cancer Cohort (4C) study. We aimed to achieve three primary objectives: (i) characterizing the absolute concentrations and composition of circulating SCFAs in normoglycemic Chinese adults; (ii) assessing the relationship between individual SCFAs objectively measured in the serum and subsequent risk of incident T2DM; and (iii) investigating whether modifiable clinical or other risk factors mediate the SCFAs-diabetes risk.

## RESULTS

### Baseline characteristics

A total of 1707 incident diabetes case subjects (1014 women and 693 men) and 1707 normal glucose regulation (NGR) individuals were included in this nested case–control study. Mean age of the total study population at baseline was 57.56 (standard deviation [SD] 8.87) years old. Baseline characteristics of participants were described previously [14, 15], and presented in Supplementary Table S1. In addition to age, sex, body mass index (BMI), and fasting plasma glucose (FPG) matched under propensity score matching, baseline fasting low-density lipoprotein cholesterol (LDL-C), smoking status, alcohol intake, and physical activity were also well-matched between case subjects and control subjects. Case subjects showed higher levels of systolic blood pressure (SBP), triglycerides (TGs), aspartate aminotransferase (AST), alanine aminotransferase (ALT), fasting insulin (FI), HOMA-IR, and HOMA of β-cell function (HOMA-B) than control subjects.

### Distribution of SCFAs and BCFAs in the study population

**Figure 1:**
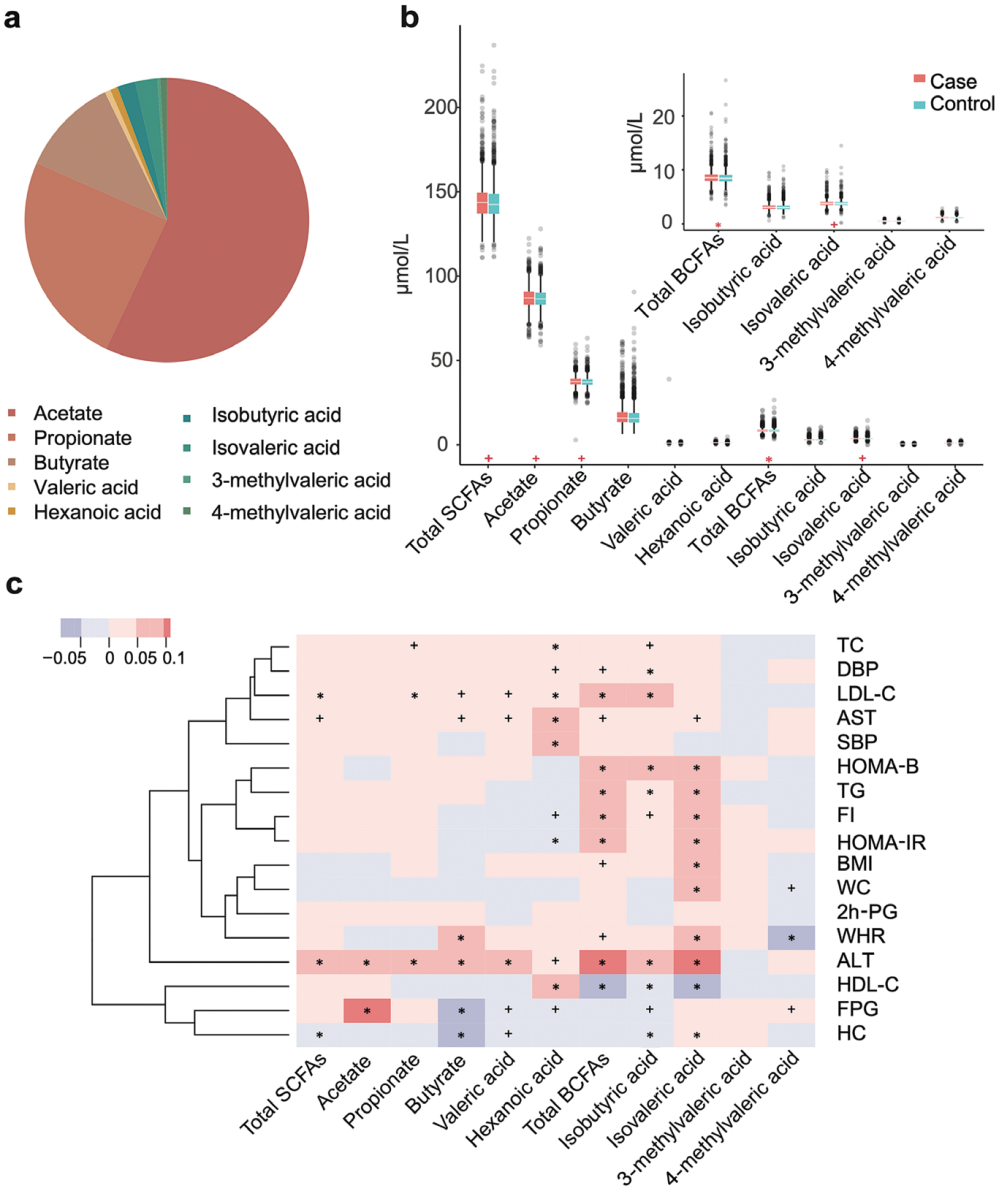
SCFA and BCFA distribution and their correlation with clinical parameters. (a) The composition of SCFAs in the total population (*n* = 3414). (b) Serum levels of SCFAs and BCFAs in case and control groups. Results are shown as boxes denoting the IQR between the first and third quartiles. The line within the boxes denotes the median. Paired Wilcoxon rank sum test, *P < 0.01, ^+^*P* < 0.05. (c) Spearman’s correlation analysis of the associations of SCFAs and BCFAs with the main clinical parameters at baseline. *P < 0.01, ^+^*P* < 0.05. The color keys represent the regression coefficients of the independent variables.

In this nested case–control study, acetate (57.0%; median [interquartile range (IQR)]: 86.77 [83.18–90.53] μmol/L), propionate (24.6%; median [IQR]: 37.40 [35.72–39.04] μmol/L), and butyrate (11.2%; median [IQR]: 15.80 [13.43–19.16] μmol/L) were the major components of serum SCFAs, whereas BCFAs were present in relatively low concentrations in the total population (Fig. 1a). Case subjects had significantly higher levels of total SCFAs, acetate, propionate, total BCFAs, and isovaleric acid than control subjects (all *P *< 0.05), whereas no difference was found in the levels of butyrate, valeric, hexanoic, isobutyric, 3-methylvaleric, and 4-methylvaleric acid between the case and control subjects (all *P *> 0.05) (Fig. 1b; Supplementary Table S2).

### Associations of SCFAs and BCFAs with the main clinical parameters at baseline

Spearman’s correlation analysis revealed that both individual and subgroup of baseline serum SCFA composition were associated with a variety of biochemical measurements and metabolic parameters in normoglycemic individuals (Fig. 1c). Of note, at baseline, both serum total BCFAs and isovaleric acid were positively associated with FI, HOMA-IR, HOMA-B, TG, ALT, AST, BMI, and waist-to-hip ratio (WHR), and inversely associated with high-density lipoprotein cholesterol (HDL-C). The sum of SCFAs showed a positive correlation with LDL-C, ALT, and AST, and a negative correlation with hip circumference (HC). Additionally, propionate also exhibited a positive association with total cholesterol (TC), LDL-C, and ALT. Taken together, these results suggest a potential relationship between circulating SCFAs and BCFAs with glucose regulation.

### Association between serum SCFAs and BCFAs with risk of incident diabetes

Conditional logistic regression models were fitted to assess the association between baseline serum SCFA and BCFA levels with future risk of diabetes in the total population and by gender (Table 1). In multivariable-adjusted models, including age, sex, BMI, smoking status, alcohol intake, physical activity, SBP, HDL-C, LDL-C, TG, AST, ALT, and FPG, per SD increment of total SCFAs and total BCFAs (especially isovaleric acid) was positively associated with incident diabetes among the overall population, with odds ratio (OR) (95% confidence interval [CI]) of 1.08 (1.00–1.17) for total SCFAs, 1.10 (1.02–1.19) for total BCFAs, and 1.10 (1.01–1.19) for isovaleric acid, respectively. However, it is important to note that the risk estimates did not retain significance after applying multiple testing correction through the false discovery rate (FDR).

**Table 1:** Associations of serum SCFA and BCFA per SD increment with risk of incident diabetes in overall population and by gender.

	Overall (*n* = 3414)		Women (*n* = 2028)		Men (*n* = 1386)		*P*_interaction_ of women and men
	OR (95% CI)	*P* value	OR (95% CI)	*P* value	OR (95% CI)	*P* value	
Model adjusting for age and sex							
Total SCFAs	1.07 (1.00–1.15)	0.06	**1.16 (1.05–1.27)**	**0.002** ^*^	0.97 (0.86–1.08)	0.55	**0.01**
Acetate	1.05 (0.98–1.13)	0.13	**1.10 (1.00–1.20)**	**0.04**	1.00 (0.90–1.11)	0.94	0.16
Propionate	1.03 (0.97–1.10)	0.38	**1.15 (1.05–1.26)**	**0.003** ^*^	0.93 (0.84–1.03)	0.14	**0.001** ^*^
Butyrate	1.05 (0.98–1.13)	0.18	1.10 (1.00–1.21)	0.05	0.99 (0.88–1.11)	0.83	0.15
Valeric acid	1.06 (0.99–1.13)	0.12	1.09 (0.99–1.20)	0.07	1.02 (0.93–1.13)	0.68	0.30
Hexanoic acid	1.07 (0.99–1.14)	0.08	**1.12 (1.02**–**1.22)**	**0.01** ^*^	0.99 (0.88–1.12)	0.89	0.09
Total BCFAs	**1.09 (1.01**–**1.17)**	**0.02**	**1.14 (1.04**–**1.26)**	**0.006** ^*^	1.02 (0.91–1.14)	0.75	0.10
Isobutyric acid	1.05 (0.97–1.12)	0.23	1.09 (0.99–1.20)	0.07	0.99 (0.89–1.11)	0.91	0.17
Isovaleric acid	**1.10 (1.02**–**1.19)**	**0.02**	**1.16 (1.04**–**1.29)**	**0.006** ^*^	1.03 (0.92–1.16)	0.60	0.11
3-methylvaleric acid	1.04 (0.97–1.11)	0.26	1.04 (0.95–1.13)	0.43	1.05 (0.94–1.17)	0.41	0.93
4-methylvaleric acid	1.05 (0.98–1.12)	0.14	1.06 (0.97–1.15)	0.21	1.05 (0.94–1.17)	0.39	0.83
Multivariable-adjusted model^†^							
Total SCFAs	**1.08 (1.00**–**1.17)**	**0.05**	**1.16 (1.05**–**1.29)**	**0.005** ^*^	0.97 (0.85–1.10)	0.64	**0.02**
Acetate	1.06 (0.99–1.15)	0.11	1.09 (0.98–1.21)	0.10	1.02 (0.91–1.15)	0.74	0.40
Propionate	1.04 (0.97–1.11)	0.26	**1.18 (1.07**–**1.31)**	**0.001** ^*^	0.92 (0.82–1.03)	0.14	**< 0.001** ^**^
Butyrate	1.04 (0.96–1.13)	0.29	1.11 (1.00–1.23)	0.06	0.97 (0.85–1.10)	0.64	0.11
Valeric acid	1.06 (0.98–1.14)	0.15	1.09 (0.99–1.22)	0.09	1.03 (0.93–1.14)	0.59	0.38
Hexanoic acid	1.06 (0.99–1.15)	0.11	1.10 (1.00–1.21)	0.06	1.00 (0.88–1.14)	1.00	0.31
Total BCFAs	**1.10 (1.02**–**1.19)**	**0.02**	**1.13 (1.02**–**1.26)**	**0.02**	1.04 (0.92–1.18)	0.49	0.23
Isobutyric acid	1.06 (0.98–1.15)	0.14	1.09 (0.98–1.22)	0.10	1.01 (0.90–1.15)	0.84	0.26
Isovaleric acid	**1.10 (1.01**–**1.19)**	**0.03**	**1.13 (1.01**–**1.26)**	**0.04**	1.05 (0.92–1.19)	0.47	0.27
3-methylvaleric acid	1.07 (0.99–1.15)	0.07	1.06 (0.96–1.17)	0.25	1.09 (0.97–1.23)	0.15	0.77
4-methylvaleric acid	1.07 (0.99–1.15)	0.08	1.06 (0.97–1.17)	0.21	1.08 (0.96–1.21)	0.22	1.00

Data in bold are statistically significant (*P* < 0.05). ^**†**^Adjusted for age, sex, BMI, smoking status, alcohol intake, physical activity, SBP, HDL-C, LDL-C, TG, AST, ALT, and FPG. *P_FDR_ < 0.05. **P_FDR_ < 0.01.

Interestingly, we observed a gender difference in the association, with strong positive associations of serum SCFAs and BCFAs with T2DM risk among women, whereas no relationship among men (Table 1). Multivariable-adjusted OR (95% CI) of per SD increment for T2DM was 1.16 (1.05–1.29) for total SCFAs, 1.18 (1.07–1.31) for propionate, 1.13 (1.02–1.26) for total BCFAs, and 1.13 (1.01–1.26) for isovaleric acid among women (*P *< 0.05). After correction by FDR, only serum total SCFAs and propionate remained significant for the risk of diabetes (FDR < 0.05). Likewise, adjusting for baseline diet score did not change the significant associations of total SCFAs and propionate with diabetes in women (Supplementary Table S3). There was a significant interaction between serum propionate and gender on the risk of developing T2DM (*P*_interaction_ < 0.001, FDR < 0.01). No significant interactions were observed between the other serum SCFAs and gender. Subgroup analysis indicated that in postmenopausal women, per SD increment in propionate was associated with a 24% higher risk of diabetes (OR = 1.24, 95% CI: 1.09–1.41, *P* = 0.001). No significant difference was observed in premenopausal women (Supplementary Table S4).

The risk for developing T2DM across SCFA quartiles is shown in Supplementary Table S5. In multivariable-adjusted logistic regression models, total SCFAs, acetate, propionate, and total BCFAs were associated with diabetes risk in overall population (*P*_trend_ < 0.05). Among women, total SCFAs, propionate, and total BCFAs were significantly associated with the risk of T2DM (*P*_trend_ < 0.05, FDR < 0.05). Using the lowest quartile as the reference, the OR (95% CI) values for T2DM in the highest quartile of total SCFAs, propionate, and total BCFAs were 1.44 (1.08–1.93), 1.58 (1.19–2.10), and 1.43 (1.07–1.90), respectively. No significant relationship between SCFAs and diabetes was observed among men.

The levels of SCFAs and BCFAs were correlated with biochemical measurements of insulin resistance and β-cell function, including HOMA-IR and HOMA-B (*P* < 0.05; Fig. 1c). Further analyses revealed that the associations of both SCFAs and BCFAs with diabetes risk were attenuated after adjustment for baseline insulin measurements, including FI, HOMA-IR, and HOMA-B in overall population (Table 2). However, the positive associations of total SCFAs and propionate with diabetes risk remained unchanged among women after adjusting for these insulin measurements. On the other hand, the associations for total BCFAs and isovaleric acid were attenuated, although directions remained unchanged (OR per SD increment: 1.08 [0.97–1.21] and 1.10 [0.99–1.24] for total BCFAs and isovaleric acid in additional adjustment of HOMA-IR, respectively).

**Table 2:** Associations of serum SCFA and BCFA levels with risk of incident diabetes in overall population and by gender, with additional adjustment for insulin measurements.

	Overall (*n* = 3414)		Women (*n* = 2028)		Men (*n* = 1386)		*P*_interaction_ of women and men
	OR (95% CI)	*P* value	OR (95% CI)	*P* value	OR (95% CI)	*P* value	
Multivariable^†^ + FI adjusted model							
Total SCFAs	1.06 (0.98–1.16)	0.15	**1.13 (1.01**–**1.26)**	**0.03**	0.97 (0.85–1.11)	0.64	0.06
Acetate	1.07 (0.99–1.16)	0.09	1.08 (0.97–1.20)	0.15	1.04 (0.92–1.19)	0.51	0.65
Propionate	1.03 (0.96–1.10)	0.46	**1.17 (1.05**–**1.31)**	**0.004** ^*^	0.90 (0.80–1.02)	0.10	**0.001** ^*^
Butyrate	1.01 (0.93–1.11)	0.74	1.07 (0.96–1.20)	0.23	0.96 (0.83–1.10)	0.52	0.16
Valeric acid	1.02 (0.94–1.10)	0.61	1.04 (0.93–1.17)	0.45	1.01 (0.90–1.12)	0.91	0.61
Hexanoic acid	1.04 (0.96–1.13)	0.37	1.07 (0.97–1.19)	0.18	0.97 (0.85–1.11)	0.66	0.32
Total BCFAs	1.06 (0.98–1.16)	0.14	1.09 (0.98–1.22)	0.12	1.01 (0.89–1.16)	0.84	0.30
Isobutyric acid	1.03 (0.94–1.12)	0.55	1.05 (0.94–1.18)	0.39	0.99 (0.87–1.13)	0.88	0.38
Isovaleric acid	1.08 (0.99–1.17)	0.08	1.11 (0.99–1.24)	0.08	1.03 (0.90–1.17)	0.71	0.30
3-methylvaleric acid	1.06 (0.98–1.15)	0.13	1.04 (0.94–1.16)	0.44	1.09 (0.96–1.24)	0.20	0.67
4-methylvaleric acid	1.05 (0.97–1.14)	0.20	1.04 (0.94–1.15)	0.43	1.06 (0.93–1.19)	0.39	0.95
Multivariable^†^ + HOMA-IR adjusted model							
Total SCFAs	1.07 (0.98–1.16)	0.13	**1.14 (1.02**–**1.27)**	**0.02**	0.97 (0.85–1.11)	0.63	**0.05**
Acetate	1.07 (0.99–1.16)	0.08	1.08 (0.98–1.21)	0.13	1.04 (0.92–1.19)	0.50	0.63
Propionate	1.03 (0.96–1.10)	0.44	**1.17 (1.05**–**1.30)**	**0.003** ^*^	0.90 (0.80–1.02)	0.10	**< 0.001** ^*^
Butyrate	1.02 (0.93–1.11)	0.69	1.08 (0.96–1.20)	0.20	0.95 (0.83–1.09)	0.50	0.14
Valeric acid	1.03 (0.95–1.11)	0.50	1.06 (0.95–1.18)	0.32	1.01 (0.90–1.12)	0.90	0.49
Hexanoic acid	1.05 (0.97–1.14)	0.26	1.09 (0.98–1.20)	0.12	0.98 (0.86–1.12)	0.74	0.29
Total BCFAs	1.06 (0.98–1.15)	0.17	1.08 (0.97–1.21)	0.14	1.01 (0.89–1.15)	0.88	0.30
Isobutyric acid	1.02 (0.94–1.11)	0.63	1.04 (0.93–1.17)	0.45	0.99 (0.87–1.12)	0.83	0.38
Isovaleric acid	1.08 (0.99–1.17)	0.08	1.10 (0.99–1.24)	0.08	1.03 (0.90–1.17)	0.70	0.31
3-methylvaleric acid	1.06 (0.98–1.15)	0.14	1.05 (0.94–1.16)	0.39	1.08 (0.95–1.23)	0.23	0.72
4-methylvaleric acid	1.05 (0.97–1.13)	0.24	1.04 (0.94–1.15)	0.47	1.05 (0.93–1.19)	0.43	0.96
Multivariable^†^ + HOMA-B adjusted model							
Total SCFAs	1.07 (0.98–1.16)	0.13	**1.14 (1.02**–**1.27)**	**0.02**	0.97 (0.85–1.11)	0.67	0.06
Acetate	1.07 (0.99–1.16)	0.09	1.08 (0.97–1.20)	0.15	1.04 (0.92–1.19)	0.50	0.65
Propionate	1.03 (0.96–1.10)	0.42	**1.17 (1.06**–**1.31)**	**0.003** ^*^	0.91 (0.80–1.02)	0.11	**0.001** ^*^
Butyrate	1.02 (0.93–1.11)	0.69	1.08 (0.96–1.20)	0.20	0.96 (0.84–1.10)	0.56	0.16
Valeric acid	1.03 (0.95–1.11)	0.48	1.06 (0.95–1.18)	0.32	1.01 (0.91–1.12)	0.87	0.53
Hexanoic acid	1.05 (0.97–1.14)	0.24	1.08 (0.98–1.20)	0.12	0.98 (0.86–1.12)	0.79	0.31
Total BCFAs	1.06 (0.98–1.15)	0.16	1.09 (0.97–1.21)	0.14	1.01 (0.89–1.15)	0.86	0.31
Isobutyric acid	1.02 (0.94–1.11)	0.62	1.04 (0.93–1.17)	0.46	0.99 (0.87–1.12)	0.86	0.41
Isovaleric acid	1.08 (0.99–1.17)	0.08	1.11 (0.99–1.24)	0.08	1.03 (0.90–1.17)	0.70	0.31
3-methylvaleric acid	1.06 (0.98–1.15)	0.14	1.05 (0.94–1.16)	0.39	1.08 (0.95–1.23)	0.23	0.72
4-methylvaleric acid	1.05 (0.97–1.13)	0.24	1.04 (0.94–1.15)	0.45	1.05 (0.93–1.18)	0.44	1.00

Data in bold are statistically significant (*P* < 0.05). ^**†**^Adjusted for age, sex, BMI, smoking status, alcohol intake, physical activity, SBP, HDL-C, LDL-C, TG, AST, ALT, and FPG. *P_FDR_ < 0.05.

### Mediation analysis between SCFAs and clinical risk factors contributing to T2DM

**Figure 2:**
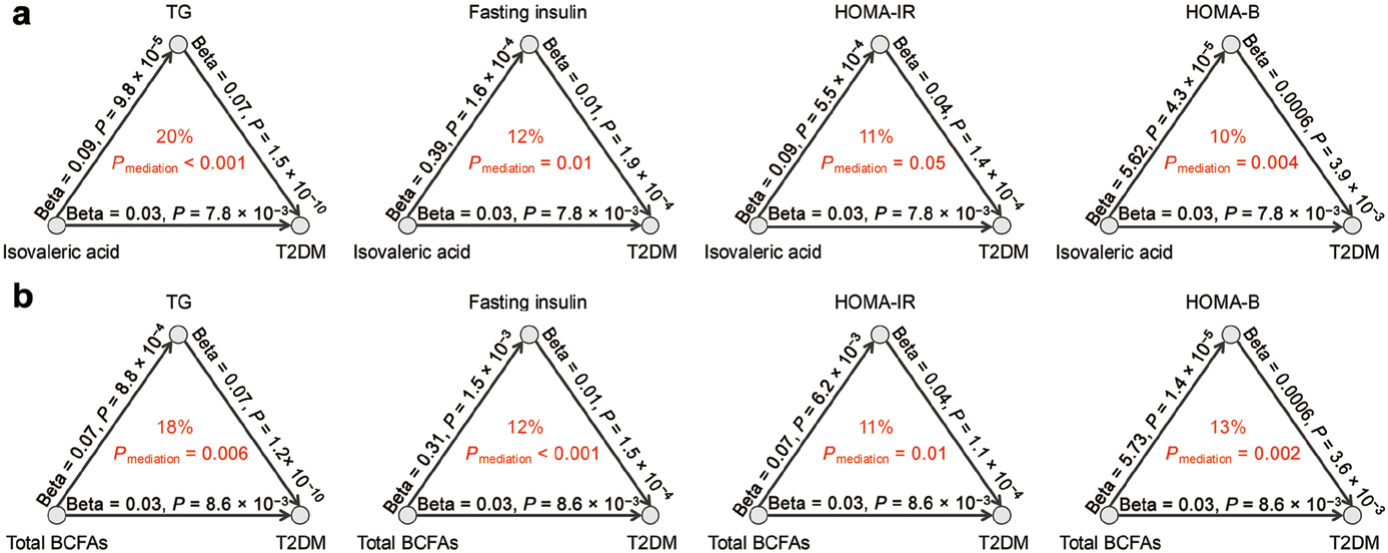
Mediation analysis among BCFAs, clinical risk factors, and T2DM in women. (a) Mediation linkages between TG/FI/HOMA-IR/HOMA-B and isovaleric acid contributed T2DM. (b) Mediation linkages between TG/FI/HOMA-IR/HOMA-B and total BCFAs contributed T2DM.

As the association between higher levels of serum SCFAs and increased T2DM risk was significant only in women, we focused our mediation analysis on this subgroup. The analysis aimed to explore the potential pathways linking clinical risk factors—including obesity, lipid metabolism, blood pressure, and glucose metabolism with the four SCFAs that were significantly associated with incident T2DM in the multivariable-adjusted models, and the onset of T2DM (Supplementary Table S6). This analysis provided deeper insights into the potential biological mechanisms through which SCFAs influence the development of T2DM in women. Eight significant mediation linkages were observed (*P*_mediation_ < 0.05; mediated proportion > 10%) (Fig. 2). TG, FI, HOMA-IR, and HOMA-B mediated 20%, 12%, 11%, and 10% of the effects of isovaleric acid on T2DM, respectively (*P*_mediation_ < 0.05 for all) (Fig. 2a). For associations between total BCFAs and incident T2DM, TG mediated 18% (*P*_mediation_ = 0.006), FI mediated 12% (*P*_mediation_ < 0.001), HOMA-IR mediated 11% (*P*_mediation_ = 0.01), and HOMA-B mediated 13% (*P*_mediation_ = 0.002) of the effect (Fig. 2b). However, the mediation effects of TG or these insulin measurements were not significant in the associations of total SCFAs and propionate with diabetes risk.

## Discussion

In the current study, we evaluated circulating SCFA and BCFA profiles in a nested case–control study drawn from a nationwide, population-based, prospective cohort of normoglycemic Chinese adults. This is the first prospective study investigating the association between circulating SCFA and BCFA profiles and the risk of developing diabetes in individuals with NGR at baseline. The findings revealed a subtle association of circulating SCFAs and BCFAs with an increased risk of incident diabetes in the total population. Notably, a positive association was identified between serum propionate and T2DM risk among women, and this association was independent of insulin measurements. In contrast, the associations of total BCFAs and isovaleric acid with incident diabetes were found to be partially mediated by insulin resistance and β-cell function.

Previous cross-sectional studies have shown differences in fecal SCFA levels between individuals with diabetes and NGR [16, 17]. One study reported predominantly reduced concentrations of fecal SCFAs, including acetate, propionate, butyrate, valeric acid, and hexanoic acid, in the T2DM patients compared with the healthy subjects [16]. Another study conducted by Zhong *et al*. also observed lower propionate level in 30 participants with T2DM compared with 30 normal controls [17]. Moreover, fecal SCFAs were negatively correlated with adiposity parameters, including BMI, visceral adipose tissue, and waist circumference (WC), confirming their beneficial effects on metabolism [18]. However, a community-based sample of Colombian adults found that higher SCFA levels in the stool are associated with indicators of higher systemic inflammation, glycemia, dyslipidemia, and obesity [19], indicating conflicting evidence on the relationship between fecal SCFAs and T2DM risk. Of note, the comparison of fecal and blood metabolome reveals their inconsistent associations with cardiometabolic diseases [20]. Fecal SCFAs reflect the balance between colonic production and absorption, which may not be generalizable to SCFAs measured in circulation [21]. Fecal SCFAs function more locally in the intestine, whereas in the circulatory system, they affect complex systemic metabolism. Therefore, the investigation into SCFA levels in the blood, which is the primary site of interaction with organs regulating blood glucose, is crucial for better understanding the potential relationship between SCFAs and metabolic health.

Previous studies have examined circulating SCFA concentrations in patients with T2DM, yielding conflicting evidence, with some suggesting higher SCFA levels and others showing lower SCFA levels in T2DM patients [12, 13]. A study of 100 Chinese individuals aged 40–60 years suggested higher serum SCFA concentrations in patients with T2DM [13]. Conversely, another study of 43 patients with T2DM and 28 overweight/obese individuals indicated that circulating SCFAs are typically low in T2DM group and inversely associated with blood glucose [12]. However, most of these studies were cross-sectional and focused on the overall population. To the best of our knowledge, this study is the first to report the associations between serum SCFAs and BCFAs with the risk of incident T2DM in a prospective cohort study, showing that higher circulating propionate is linked to an increased risk of developing diabetes among women with NGR.

In agreement with our findings, previous research in both humans and mouse models indirectly supports a detrimental rather than a beneficial metabolic effect of circulating propionate and isovaleric acid on glucose regulation. Specially, in obesity-prone subjects, there is a significantly higher concentration of plasma and fecal propionate, and the gut microbiota-derived propionate induces hypermethylation in the promoter region of disabled-1 (*DAB1*), thereby increasing the susceptibility to diabetes [22]. Additionally, Mendelian randomization analyses have causally linked abnormalities in the production or absorption of fecal propionate to an increased risk of T2DM (*P *= 0.004) [23]. With regards to isovaleric acid, higher proportions have been detected in overweight individuals compared to those with normal weight, and there was a positive correlation with worsened blood lipid parameters and inflammatory cytokines [6, 24, 25]. It is possible that a high serum concentration of isovaleric acid is a marker for a poor metabolism status. In our cohort, the positive association between per SD increment of serum isovaleric acid and an increased diabetes risk diminished after multiple tests for FDR. Further studies are necessary to unravel the detailed mechanisms through which propionate and isovaleric acid regulate glucose metabolism.

Metabolic disparities between males and females are widely recognized, and our findings have suggested a significant association of serum SCFA concentration with the development of diabetes in women, particularly postmenopausal women, but not in men. However, the underlying mechanisms contributing to this sex-based divergence remain unclear. Hormonal variations, lifestyle factors, and dietary habits are all capable of modulating the composition and abundance of gut microbiota [26], which in turn may exert an impact on human health and disease via the production of harmful or beneficial metabolites [27]. SCFAs, the principle fermentation products of gut microbiota [28], have been implicated in glucose metabolism. Our previous study has reported sexual dimorphisms in gut microbiota, with male mice exhibiting a greater abundance of *Prevotella*, while female mice displaying a higher abundance of *Akkermansia muciniphila* [29]. These sex hormone-mediated disparities in the gut microbiome drive sex bias in circulating metabolites, which are associated with glucose metabolism [29]. Additional investigations are needed to determine whether the effects of SCFAs on glucose metabolism are contingent upon the sex hormones or gut microbiome.

Impaired insulin secretion and insulin resistance are recognized as pivotal factors in the pathogenesis of T2DM. Elevated levels of circulating BCAAs have been reported to be associated with future insulin resistance and T2DM [15, 30]. BCFAs as one of the metabolism productions of BCAAs are likely to link with insulin resistance, and SCFAs from the fermentation of resistant dietary carbohydrates are known modulators of insulin secretion [31]. Previous studies indicated that isovaleric acid can activate mammalian target of rapamycin complex 1/S6 kinase 1 (mTORC1/S6K1) in hepatocytes, leading to elevated gluconeogenesis and the development of insulin resistance [8]. Tirosh *et al*. found that long-term exposure of mice to a daily low dose of propionate results in a gradual weight gain and insulin resistance, and in human, consumption of a propionate-containing mixed meal results in a postprandial increase in plasma glucagon, fatty acid-binding protein 4, and norepinephrine, leading to insulin resistance and compensatory hyperinsulinemia [32]. Interestingly, our study provide further evidence from the large prospective cohort. We demonstrated that serum SCFA (i.e. propionate) might be a potential predictive factor for diabetes independent of insulin measurements, including FI, HOMA-IR, and HOMA-B, whereas serum BCFA (i.e. isovaleric acid) could promote diabetes risk via hyperinsulinemia, insulin resistance, or β-cell dysfunction. The precise causal links between the above observations await further mechanistic elucidation. Taken together, our results support the notion that serum SCFAs and BCFAs might be implicated in the early pathogenesis of glucose dysregulation, preceding the clinical onset of diabetes.

This study has several important strengths, including the large sample size from a nationwide population-based prospective cohort study design, the population being normoglycemic at baseline, accurate measurement of the levels of nine different serum SCFAs and BCFAs by using gas chromatography-mass spectrometry (GC-MS), and comprehensive adjustment for various diabetes risk factors, including lifestyles, body composition, liver function, blood pressure, and lipid metabolism. It is worth mentioning that the measurement of the glucose regulation status was based on the oral glucose tolerance test (OGTT) at both baseline and the follow-up visits, which makes it possible for accurate evaluation of glucose regulation status. Moreover, our study includes a comprehensive evaluation of potential mediation pathways and investigation of gender intercorrelations.

In conclusion, our findings provide valuable insights into the association between circulating SCFAs and BCFAs with the risk of T2DM from a large nationwide prospective cohort. The gender-specific association with propionate emphasizes the importance of considering sex-specific factors in understanding diabetes susceptibility.

### Limitations of the study

Our study has several limitations that merit attention. First, the 3-year follow-up period was relatively short, and longer follow-up periods are needed to confirm the results. Second, only serum SCFAs were measured, not with fecal samples, limiting further insights into the association between gut microbiota, SCFAs, and metabolic outcomes. Third, serum SCFAs might reflect both dietary intake and endogenous synthesis, which are affected by complex exogenous and genetic factors. Due to a lack of detailed diet information and gene analysis from participants, these factors could not be fully elucidated in this study. Fourth, the relatively small number of men with a short follow-up duration and missing information on sex hormones might limit the ability to provide precise estimates, hindering further exploration of the absence of significant associations in men. Fifth, baseline metabolite measurements were conducted only once in this cohort, along with two-point blood glucose measurements at baseline and follow-up. This restricted our ability to effectively use more advanced analytical methods, such as linear mixed models. Finally, the study population comprises Chinese individuals aged ≥ 40 years, and therefore, the findings may not be generalizable to other ethnic and age groups.

## Materials and methods

### Study population

The 4C study is a nationwide, population-based, prospective cohort study. At baseline, 193,846 adults aged ≥ 40 years were recruited from resident registration systems of 20 communities in China during 2011–2012. All participants were invited to attend an in-person follow-up visit between 2014 and 2016, and 170,240 (87.8%) participants had available follow-up data. The methods of the 4C project have previously been described in detail [33, 34].

To summarize, we conducted a nested case–control study of 1707 (1014 women and 693 men) matched case subject–control subject pairs within the 4C study, as previously described [14, 15]. From 54,807 subjects defined as having NGR based on a 75-g OGTT at baseline, we identified 1864 participants who developed T2DM during a median follow-up of 3.03 (IQR: 2.87–3.24) years. After exclusions for 157 participants without baseline serum samples, 1707 incident case subjects with diabetes remained in the current study. The control group of 1707 NGR individuals at baseline was selected using propensity score matching with a logistic model that included age, sex, BMI, and FPG [35].

The study protocol was approved by the Institutional Review Board of Ruijin Hospital affiliated to Shanghai Jiao Tong University School of Medicine. Informed consent was obtained from study participants.

### Data collection

At baseline and follow-up visits, information on demographic characteristics, lifestyle factors, and medical history was collected in local community clinics by trained study personnel according to a standardized questionnaire. Smoking status and alcohol drinking were categorized as never, former, or current. The International Physical Activity Questionnaire was used to assess physical activity [36]. Moderate or vigorous physical activity was defined as ≥ 150 min/week of moderate-intensity physical activity, 75 min/week of vigorous aerobic activity, or an equivalent combination of moderate-intensity and vigorous aerobic activities. A semi-quantitative Chinese Food Frequency Questionnaire was used to collect habitual dietary intake during the previous 12 months, and a diet score was calculated according to the recommendation of the American Heart Association with replacement of whole grains with bean consumption [37]. Height, weight, WC, and HC were measured according to a standard protocol. BMI was calculated as the weight in kilograms divided by height in meters squared. Three measurements of blood pressure obtained by using an automated electronic device (Model HEM-752 FUZZY; Omron Healthcare, Dalian, China) in a seated position after at least a 5-min quiet rest were averaged for analysis.

Blood samples were collected in all participants after an overnight fast of at least 10 h. Sera were shipped by air in dry ice to the central laboratory and stored at −80°C in the Ruijin Biobank. Levels of LDL-C, HDL-C, TGs, ALT, and AST were measured with an autoanalyser (ARCHITECT c16000 System, Abbott Laboratories, IL, USA) in the central laboratory located at Shanghai Institute of Endocrine and Metabolic Diseases, which was certified by the College of American Pathologists. Insulin resistance was estimated by the HOMA-IR index: fasting insulin (µIU/mL) × fasting glucose (mg/dL)/405 [38]. β-cell function was estimated by the HOMA-B index: (360 × fasting insulin [µIU/mL])/(fasting glucose [mg/dL] − 63) [38].

### Definition of diabetes

At both baseline and follow-up visits, the concentrations of FPG and 2-h plasma glucose (2h-PG) were collected at 0 and 2 h during the 75-g OGTT and measured using the glucose oxidase or hexokinase method. Incident diabetes was defined as FPG of 126 mg/dL or more, 2h-PG of 200 mg/dL or more, or self-reported previous diagnosis of diabetes by physicians and the current use of antidiabetic medications.

### SCFA and BCFA measurements

The baseline serum SCFAs (acetate, propionate, butyrate, valeric acid, and hexanoic acid) and BCFAs (isobutyric acid, isovaleric acid, 3-methylvaleric acid, and 4-methylvaleric acid) were measured using an Agilent 5977A GC-MS system (Agilent, USA), fitted with a DB-FFAP column (30 m × 250 μm × 0.25 μm, Agilent, USA). The standard solutions of SCFAs were prepared at 0.0002–3.33 μmol/L. The thawed serum (30 μL) was mixed with 60 μL acetonitrile containing 0.3 μg/mL C4-d7 as the internal standard. Samples were further vortexed and centrifuged. The chromatography was programmed for an initial temperature of 50°C for 1 min. The temperature was increased to 180°C at 10 °C/min, increased to 250°C at 30 °C/min, and then held for 2 min. The flow rate of helium carrier gas was kept at 40 cm/s. The temperatures of injector, ion source, quadrupole, and interface were 250°C, 230°C, 150°C, and 250°C, respectively. The selected ion monitoring mode was performed by detecting the protonated molecules at *m/z* 43 for acetate and isobutyric acid, *m/z* 74 for propionate, and *m/z* 60 for other SCFAs and BCFAs. The identification of each SCFA was based on the retention time and corresponding mass spectra of reference compounds, with the assistance of standard reference materials. Total SCFA concentration was the sum of levels of acetate, propionate, butyrate, valeric acid, and hexanoic acid, and total BCFA concentration was the sum of levels of isobutyric acid, isovaleric acid, 3-methylvaleric acid, and 4-methylvaleric acid.

### Statistical analysis

Baseline characteristics of participants are presented as means (SDs) or medians (IQR) for continuous variables with or without normal distribution, respectively, and numbers (proportions) for categorical variables. We compared cases (those developing new-onset diabetes during the 3.03-year follow-up) versus propensity-matched controls, using paired Wilcoxon rank sum test for the serum SCFAs at baseline. Spearman’s correlation analysis was used to assess the associations of individual SCFA with the main clinical parameters, including BMI, WC, HC, WHR, SBP, diastolic blood pressure (DBP), FPG, 2h-PG, FI, HOMA-IR, HOMA-B, ALT, AST, and lipids at baseline.

Serum SCFAs were log-transformed before analysis to ensure normality. To explore the dose–effect relationship, we conducted quartile analyses using cutoff values derived from control subjects. OR and 95% CI values of developing T2DM represented as both quartiles and per SD increase in each SCFA were calculated by conditional (matched-pairs) logistic regression analysis. We adjusted for potential confounders, including age, sex, BMI, smoking status, alcohol intake, physical activity, and clinical parameters (SBP, HDL-C, LDL-C, TG, AST, ALT, and FPG). Further analyses included additional adjustments for FI, HOMA-IR, or HOMA-B to investigate whether the predictive effect of serum SCFAs on diabetes risk was independent of insulin resistance and β-cell function. *P* value was corrected for multiple testing via FDR using the Benjamini–Hochberg method. To test whether the pattern of association between SCFAs and T2DM varies across stratifications, we stratified participants into subgroups according to sex and by the menopause status in women. In the sensitivity analyses, we analyzed the effects of dietary habits on the observed associations.

Mediation analysis was conducted using the R package “mediation” to examine potential mediation relationships between SCFAs and T2DM. Models of mediation analysis were formulated as follows (Y as outcome-incident T2DM, X as exposure-SCFA, M as mediation-clinical risk factors): Y = cX + e1, M = aX + e2, and Y = c′X + bM + e3, where c = c′ + ab, c as total effect of SCFA, c′ as direct effect of SCFA, and ab as indirect effect of SCFA mediated by risk factor. Relationship groups which meet the following criteria were defined as significant mediation relationships: (ⅰ) the total effect of SCFA on T2DM is significant (*P*_total effect_ < 0.05); (ⅱ) mediated proportion (c′/c) > 10%; and (ⅲ) the indirect effect is significant (*P*_mediation_ < 0.05) [39].

A two-tailed *P* value < 0.05 was considered statistically significant. Statistical analyses were performed using R 4.2.3 software.

## Supplementary Material

loaf001_suppl_Supplementary_Material

## Data Availability

The authors confirm that all the data supporting the findings of this study are available within the supplementary material and corresponding authors.
