# Supplementary material for: Circulating short-chain and branched short-chain fatty acids and the risk of incident type 2 diabetes: findings from the 4C study
Source: Life Metab. 2025 Jan 22;4(2):loaf001. doi: 10.1093/lifemeta/loaf001 (PMC11897982; doi:10.1093/lifemeta/loaf001)
Supplement: loaf001_suppl_Supplementary_Material [file loaf001_suppl_supplementary_material.docx]

**Supplementary Material**

**Supplementary Table S1** Baseline characteristics of the nested case and control subjects from the 4C study.

|  | Case subjects (*n* = 1,707) | Control subjects (*n* = 1,707) | *P* value |
| --- | --- | --- | --- |
| Age years | 57.8 ± 8.9 | 57.3 ± 8.9 | 0.14 |
| Male sex | 693 (40.6) | 693 (40.6) | 1.00 |
| BMI, kg/m^2^ | 24.9 ± 3.7 | 24.7 ± 3.5 | 0.05 |
| Hip circumference, cm | 95.5 ± 8.0 | 95.2 ± 8.1 | 0.30 |
| Moderate and vigorous physical activity | 235 (13.8) | 222 (13.0) | 0.11 |
| Current smoker | 300 (17.6) | 323 (18.9) | 0.33 |
| Current drinker | 196 (11.5) | 213 (12.5) | 0.40 |
| SBP, mmHg | 136.9 ± 21.5 | 133.4 ± 20.7 | < 0.0001 |
| FPG, mmol/L | 5.43 ± 0.42 | 5.40 ± 0.40 | 0.07 |
| 2h-PG, mmol/L | 6.27 ± 1.10 | 5.98 ± 1.15 | < 0.0001 |
| Fasting insulin, uIU/mL | 6.70 (4.50−9.80) | 6.33 (4.60−8.60) | < 0.0001 |
| HOMA-IR | 1.86 ± 1.15 | 1.69 ± 0.89 | < 0.0001 |
| HOMA-B | 83.54 ± 64.32 | 76.02 ± 47.61 | 0.0002 |
| Fasting TGs, mmol/L | 1.34 (0.95−1.98) | 1.19 (0.86−1.67) | < 0.0001 |
| Fasting HDL cholesterol, mmol/L | 1.33 ± 0.38 | 1.36 ± 0.36 | 0.007 |
| Fasting LDL cholesterol, mmol/L | 2.89 ± 0.90 | 2.86 ± 0.85 | 0.31 |
| Fasting cholesterol, mmol/L | 4.99 ± 1.15 | 4.92 ± 1.09 | 0.02 |
| AST, IU/L | 23.1 ± 13.9 | 21.9 ± 9.8 | < 0.0001 |
| ALT, IU/L | 18.3 ± 13.6 | 16.6 ± 11.7 | < 0.0001 |

Values are *n* (%), mean ± SD, or median (interquartile range).

**Supplementary Table S2** Paired Wilcoxon *P* values of SCFAs in the two groups, related to Figure 1.

| Biochemical (umol/L) | Case subjects (*n* = 1,707) | Control subjects (*n* = 1,707) | *P* value |
| --- | --- | --- | --- |
| Total SCFAs | 143.55 (137.19−149.46) | 142.49 (137.01−148.71) | 0.021 |
| Acetate | 87.11 (83.18−90.83) | 86.53 (83.19−90.25) | 0.047 |
| Propionate | 37.54 (35.86−39.145) | 37.26 (35.61−38.92) | 0.050 |
| Butyrate | 15.90 (13.61−19.42) | 15.66 (13.29−18.99) | 0.146 |
| Valeric acid | 1.00 (0.92−1.09) | 1.00 (0.91−1.09) | 0.234 |
| Hexanoic acid | 1.30 (1.20−1.44) | 1.29 (1.19−1.42) | 0.051 |
| Total BCFAs | 8.53 (7.98−9.14) | 8.43 (7.90−9.06) | 0.008 |
| Isobutyric acid | 3.07 (2.76−3.42) | 3.03 (2.71−3.40) | 0.082 |
| Isovaleric acid | 3.77 (3.50−4.06) | 3.74 (3.49−4.02) | 0.026 |
| 3-methylvaleric acid | 0.50 (0.45−0.55) | 0.50 (0.45−0.55) | 0.503 |
| 4-methylvaleric acid | 1.13 (1.00−1.3) | 1.12 (0.99−1.27) | 0.255 |

**Supplementary Table S3** Sensitivity analysis: associations of serum SCFA and BCFA levels with risk of incident diabetes in overall population and by gender, with additional adjustment for diet score.

|  | Overall  (*n* = 3,414) | | Women  (*n* = 2,028) | | Men  (*n* = 1,386) | | *P*_interaction_ of women and men |
| --- | --- | --- | --- | --- | --- | --- | --- |
|  | OR (95% CI) | *P* value | OR (95% CI) | *P* value | OR (95% CI) | *P* value |  |
| Total SCFAs | 1.08 (0.99−1.18) | 0.10 | **1.18 (1.05−1.33)** | **0.005^*^** | 0.94 (0.81−1.08) | 0.36 | **0.01** |
| Acetate | 1.04 (0.95−1.14) | 0.37 | 1.08 (0.96−1.21) | 0.23 | 0.98 (0.85−1.13) | 0.78 | 0.40 |
| Propionate | 1.04 (0.96−1.12) | 0.37 | **1.19 (1.06−1.34)** | **0.003^*^** | 0.92 (0.81−1.04) | 0.17 | **0.003^*^** |
| Butyrate | 1.06 (0.97−1.16) | 0.21 | **1.14 (1.01−1.29)** | **0.03** | 0.95 (0.82−1.10) | 0.52 | **0.05** |
| Valeric acid | 1.07 (0.97−1.17) | 0.17 | **1.13 (1.00−1.28)** | **0.04** | 1.00 (0.86−1.15) | 0.95 | 0.16 |
| Hexanoic acid | 1.09 (0.99−1.19) | 0.07 | **1.14 (1.02−1.29)** | **0.03** | 1.02 (0.88−1.17) | 0.82 | 0.24 |
| Total BCFAs | **1.09 (1.00−1.19)** | **0.05** | **1.13 (1.00−1.27)** | **0.04** | 1.03 (0.89−1.19) | 0.70 | 0.23 |
| Isobutyric acid | 1.08 (0.99−1.18) | 0.09 | 1.13 (1.00−1.27) | 0.05 | 1.01 (0.88−1.16) | 0.86 | 0.16 |
| Isovaleric acid | 1.07 (0.97−1.18) | 0.19 | 1.10 (0.97−1.26) | 0.15 | 1.00 (0.85−1.17) | 0.97 | 0.23 |
| 3-methylvaleric acid | 1.05 (0.96−1.14) | 0.28 | 1.02 (0.91−1.14) | 0.73 | 1.11 (0.97−1.27) | 0.14 | 0.28 |
| 4-methylvaleric acid | 1.07 (0.98−1.16) | 0.14 | 1.07 (0.96−1.19) | 0.25 | 1.07 (0.94−1.23) | 0.30 | 0.96 |

Data in bold are statistically significant (*P* < 0.05). Models were adjusted for age, sex, BMI, smoking status, alcohol intake, physical activity, diet, SBP, HDL-C, LDL-C, TG, AST, ALT, and FPG. ^*^*P*_FDR_ < 0.05.

**Supplementary Table S4** Association of serum propionate per SD increment with risk of incident diabetes in women with different menopause status^*^.

|  | Pre-menopausal women  (*n* = 397) | | Post-menopausal women  (*n* = 1,490) | | *P*_interaction_ |
| --- | --- | --- | --- | --- | --- |
|  | OR (95% CI) | *P* value | OR (95% CI) | *P* value |  |
| Model adjusting for age | 1.12 (0.78−1.61) | 0.53 | **1.16 (1.03−1.30)** | **0.01** | 0.52 |
| Multivariable-adjusted model^†^ | 0.89 (0.43−1.84) | 0.76 | **1.24 (1.09−1.41)** | **0.001** | 0.28 |
| Multivariable^†^ + FI adjusted model | 1.05 (0.44−2.48) | 0.91 | **1.25 (1.09−1.43)** | **0.001** | 0.34 |
| Multivariable^†^ + HOMA-IR adjusted model | 1.05 (0.44−2.47) | 0.92 | **1.24 (1.09−1.42)** | **0.001** | 0.39 |
| Multivariable^†^ + HOMA-B adjusted model | 1.05 (0.44−2.51) | 0.91 | **1.24 (1.09−1.42)** | **0.001** | 0.37 |

Data in bold are statistically significant (*P* < 0.05).

^*^A total of 1,887 participants (397 pre-menopausal and 1,490 post-menopausal women) were included in the analysis.

^†^Adjusted for age, BMI, smoking status, alcohol intake, physical activity, SBP, HDL-C, LDL-C, TG, AST, ALT, and FPG.

**Supplementary Table S5** Association of serum SCFA and BCFA levels with risk of T2DM in overall population and by gender.

|  | Quartiles of SCFAs | | | | |
| --- | --- | --- | --- | --- | --- |
|  | Quartile 1 | Quartile 2 | Quartile 3 | Quartile 4 | *P*_trend_ |
| Overall | | | | | |
| Total SCFAs | 1.00 (ref) | 0.90 (0.72−1.11) | 1.21 (0.98−1.50) | 1.16 (0.93−1.44) | **0.04** |
| Acetate | 1.00 (ref) | 0.89 (0.72−1.11) | 1.05 (0.85−1.29) | 1.22 (0.98−1.52) | **0.03** |
| Propionate | 1.00 (ref) | 1.04 (0.84−1.28) | 1.20 (0.97−1.48) | **1.28 (1.03−1.58)** | **0.01** |
| Butyrate | 1.00 (ref) | 1.17 (0.94−1.45) | 1.23 (0.99−1.53) | 1.20 (0.96−1.51) | 0.11 |
| Valeric acid | 1.00 (ref) | **1.25 (1.01−1.55)** | 1.18 (0.95−1.47) | 1.12 (0.89−1.40) | 0.54 |
| Hexanoic acid | 1.00 (ref) | 1.18 (0.95−1.46) | 1.02 (0.82−1.27) | 1.16 (0.94−1.43) | 0.35 |
| Total BCFAs | 1.00 (ref) | 1.10 (0.88−1.37) | 1.21 (0.98−1.50) | **1.33 (1.07−1.65)** | **0.006** |
| Isobutyric acid | 1.00 (ref) | 1.17 (0.94−1.46) | 1.19 (0.95−1.48) | 1.25 (1.00−1.56) | 0.06 |
| Isovaleric acid | 1.00 (ref) | 0.99 (0.80−1.23) | 0.95 (0.78−1.17) | 1.19 (0.97−1.48) | 0.14 |
| 3-methylvaleric acid | 1.00 (ref) | 1.08 (0.87−1.34) | 1.08 (0.87−1.34) | 1.13 (0.92−1.39) | 0.27 |
| 4-methylvaleric acid | 1.00 (ref) | 1.02 (0.82−1.27) | 1.02 (0.83−1.27) | 1.14 (0.93−1.40) | 0.22 |
| Women | | | | | |
| Total SCFAs | 1.00 (ref) | 1.00 (0.75−1.33) | **1.39 (1.05−1.86)** | **1.44 (1.08−1.93)** | **0.003^*^** |
| Acetate | 1.00 (ref) | 0.75 (0.56−1.01) | 1.16 (0.88−1.53) | 1.15 (0.86−1.53) | 0.08 |
| Propionate | 1.00 (ref) | 1.18 (0.88−1.57) | **1.60 (1.20−2.14)** | **1.58 (1.19−2.10)** | **<0.001^*^** |
| Butyrate | 1.00 (ref) | **1.48 (1.10−2.00)** | **1.63 (1.21−2.18)** | **1.43 (1.05−1.93)** | **0.03** |
| Valeric acid | 1.00 (ref) | 1.14 (0.86−1.52) | 1.20 (0.91−1.59) | 1.19 (0.89−1.59) | 0.23 |
| Hexanoic acid | 1.00 (ref) | **1.43 (1.07−1.91)** | 1.14 (0.85−1.53) | **1.34 (1.01−1.77)** | 0.17 |
| Total BCFAs | 1.00 (ref) | 1.01 (0.75−1.36) | 1.21 (0.92−1.60) | **1.43 (1.07−1.90)** | **0.006^*^** |
| Isobutyric acid | 1.00 (ref) | 1.21 (0.91−1.60) | **1.34 (1.01−1.79)** | 1.28 (0.96−1.72) | 0.07 |
| Isovaleric acid | 1.00 (ref) | 0.95 (0.72−1.27) | 0.97 (0.74−1.28) | 1.26 (0.95−1.65) | 0.10 |
| 3-methylvaleric acid | 1.00 (ref) | 1.09 (0.82−1.45) | 0.95 (0.71−1.28) | 1.08 (0.82−1.43) | 0.79 |
| 4-methylvaleric acid | 1.00 (ref) | 1.01 (0.76−1.34) | 0.92 (0.69−1.22) | 1.08 (0.81−1.43) | 0.75 |
| Men | | | | | |
| Total SCFAs | 1.00 (ref) | 0.90 (0.65−1.25) | 1.01 (0.72−1.41) | 0.89 (0.63−1.27) | 0.70 |
| Acetate | 1.00 (ref) | 0.92 (0.65−1.28) | 0.93 (0.67−1.30) | 1.18 (0.84−1.65) | 0.37 |
| Propionate | 1.00 (ref) | 0.92 (0.66−1.28) | 0.86 (0.61−1.20) | 0.98 (0.70−1.38) | 0.78 |
| Butyrate | 1.00 (ref) | 0.98 (0.71−1.36) | 0.84 (0.59−1.19) | 1.02 (0.72−1.44) | 0.90 |
| Valeric acid | 1.00 (ref) | 1.26 (0.90−1.77) | 1.20 (0.86−1.69) | 0.90 (0.64−1.27) | 0.49 |
| Hexanoic acid | 1.00 (ref) | 0.93 (0.67−1.29) | 0.89 (0.63−1.26) | 0.95 (0.69−1.32) | 0.73 |
| Total BCFAs | 1.00 (ref) | 1.29 (0.92−1.80) | 1.24 (0.87−1.75) | 1.13 (0.80−1.61) | 0.59 |
| Isobutyric acid | 1.00 (ref) | 1.07 (0.76−1.51) | 1.07 (0.75−1.51) | 1.16 (0.82−1.65) | 0.43 |
| Isovaleric acid | 1.00 (ref) | 1.12 (0.80−1.58) | 0.95 (0.67−1.34) | 1.10 (0.79−1.54) | 0.82 |
| 3-methylvaleric acid | 1.00 (ref) | 1.10 (0.79−1.54) | 1.26 (0.91−1.76) | 1.21 (0.87−1.66) | 0.20 |
| 4-methylvaleric acid | 1.00 (ref) | 1.13 (0.80−1.59) | 1.02 (0.72−1.44) | 1.31 (0.94−1.80) | 0.15 |

Data in bold are statistically significant (*P* < 0.05). Models were adjusted for age, sex, BMI, smoking status, alcohol intake, physical activity, SBP, HDL-C, LDL-C, TG, AST, ALT, and FPG. ^*^*P*_FDR_ < 0.05.

**Supplementary Table S6** Results of causal mediation analysis, related to Figure 2.

| SCFA | Clinical risk factor | β_a_ | *P*_a_ | β_b_ | *P*_b_ | β_c_ | *P*_c_ | β_acme_ | *P*_acme_ | β_ade_ | *P*_ade_ | β_total effect_ | *P*_total effect_ | Mediated prop. | *P*_Mediated prop_ |
| --- | --- | --- | --- | --- | --- | --- | --- | --- | --- | --- | --- | --- | --- | --- | --- |
| Total BCFAs | FI | 0.313 | 1.49E−03 | 0.01 | 1.49E−04 | 0.029 | 0.009 | 0.003 | 0.00E+00 | 0.024 | 0.04 | 0.027 | 0.014 | 0.119 | 0.014 |
|  | HOMA-IR | 0.066 | 0.006 | 0.042 | 1.11E−04 | 0.029 | 0.009 | 0.003 | 0.012 | 0.023 | 0.05 | 0.026 | 0.036 | 0.107 | 0.048 |
|  | HOMA-B | 5.732 | 1.41E−05 | 0.001 | 3.63E−03 | 0.029 | 0.009 | 0.003 | 2.00E−03 | 0.023 | 0.058 | 0.026 | 0.036 | 0.128 | 0.038 |
|  | Hypertension | 0.008 | 0.458 | 0.085 | 1.44E−04 | 0.029 | 0.009 | 0.001 | 0.436 | 0.029 | 0.008 | 0.029 | 0.008 | 0.024 | 0.436 |
|  | Dyslipidemia | 0.029 | 0.007 | 0.094 | 4.64E−05 | 0.029 | 0.009 | 0.003 | 0.006 | 0.027 | 0.02 | 0.029 | 0.008 | 0.093 | 0.014 |
|  | TG | 0.073 | 8.75E−04 | 0.073 | 1.21E−10 | 0.029 | 0.009 | 0.005 | 0.006 | 0.025 | 0.03 | 0.03 | 0.008 | 0.177 | 0.01 |
|  | TC | 0.085 | 1.71E−03 | 0.021 | 0.021 | 0.029 | 0.009 | 0.002 | 0.014 | 0.027 | 0.02 | 0.029 | 0.016 | 0.062 | 0.03 |
|  | LDL-C | 0.066 | 9.49E−04 | 0.021 | 0.089 | 0.029 | 0.009 | 0.001 | 0.082 | 0.027 | 0.022 | 0.029 | 0.016 | 0.049 | 0.094 |
|  | HDL-C | −0.014 | 0.095 | −0.092 | 2.77E−03 | 0.029 | 0.009 | 0.001 | 0.08 | 0.027 | 0.026 | 0.029 | 0.016 | 0.044 | 0.096 |
|  | Abdominal obesity | 0.014 | 0.212 | 0.059 | 0.009 | 0.029 | 0.009 | 0.001 | 0.208 | 0.03 | 0.018 | 0.03 | 0.014 | 0.027 | 0.222 |
|  | Obesity | 0.004 | 0.647 | 0.065 | 0.031 | 0.029 | 0.009 | 0 | 0.692 | 0.029 | 0.008 | 0.029 | 0.008 | 0.008 | 0.692 |
|  | BMI | 0.054 | 0.527 | 0.003 | 0.281 | 0.029 | 0.009 | 0 | 0.642 | 0.029 | 0.008 | 0.029 | 0.008 | 0.006 | 0.642 |
|  | WC | −0.061 | 0.784 | 0.002 | 0.166 | 0.029 | 0.009 | 0 | 0.856 | 0.03 | 4.00E−03 | 0.03 | 0.008 | −0.003 | 0.86 |
|  | HC | −0.208 | 0.255 | −0.001 | 0.668 | 0.029 | 0.009 | 0 | 0.734 | 0.03 | 0.01 | 0.03 | 0.01 | 0.004 | 0.732 |
|  | WHR | 0.001 | 0.421 | 0.453 | 4.91E−03 | 0.029 | 0.009 | 0.001 | 0.42 | 0.029 | 0.006 | 0.03 | 0.006 | 0.019 | 0.422 |
| Isovaleric acid | FI | 0.386 | 1.60E−04 | 0.01 | 1.86E−04 | 0.031 | 0.008 | 0.004 | 0.012 | 0.028 | 0.01 | 0.032 | 2.00E−03 | 0.121 | 0.014 |
|  | HOMA-IR | 0.087 | 5.46E−04 | 0.041 | 1.43E−04 | 0.031 | 0.008 | 0.004 | 0.046 | 0.028 | 0.014 | 0.032 | 4.00E−03 | 0.114 | 0.05 |
|  | HOMA-B | 5.616 | 4.30E−05 | 0.001 | 3.91E−03 | 0.031 | 0.008 | 0.003 | 4.00E−03 | 0.028 | 0.01 | 0.032 | 4.00E−03 | 0.102 | 0.008 |
|  | Hypertension | −0.003 | 0.794 | 0.086 | 1.13E−04 | 0.031 | 0.008 | 0 | 0.83 | 0.031 | 0.008 | 0.031 | 0.008 | −0.008 | 0.83 |
|  | Dyslipidemia | 0.028 | 0.011 | 0.094 | 4.50E−05 | 0.031 | 0.008 | 0.003 | 0.016 | 0.028 | 0.014 | 0.031 | 0.008 | 0.086 | 0.024 |
|  | TG | 0.089 | 9.84E−05 | 0.073 | 1.49E−10 | 0.031 | 0.008 | 0.006 | 0.00E+00 | 0.025 | 0.038 | 0.032 | 4.00E−03 | 0.203 | 4.00E−03 |
|  | TC | 0.061 | 0.033 | 0.022 | 0.018 | 0.031 | 0.008 | 0.001 | 0.038 | 0.03 | 0.012 | 0.032 | 0.006 | 0.042 | 0.044 |
|  | LDL-C | 0.042 | 0.043 | 0.022 | 0.078 | 0.031 | 0.008 | 0.001 | 0.11 | 0.03 | 0.014 | 0.031 | 0.01 | 0.03 | 0.116 |
|  | HDL-C | −0.018 | 0.032 | −0.091 | 3.05E−03 | 0.031 | 0.008 | 0.002 | 0.014 | 0.03 | 0.014 | 0.031 | 0.01 | 0.053 | 0.024 |
|  | Abdominal obesity | 0.025 | 0.028 | 0.058 | 0.011 | 0.031 | 0.008 | 0.001 | 0.04 | 0.03 | 0.01 | 0.032 | 0.008 | 0.046 | 0.048 |
|  | Obesity | 0.006 | 0.489 | 0.065 | 0.032 | 0.031 | 0.008 | 0 | 0.61 | 0.031 | 0.01 | 0.031 | 0.008 | 0.012 | 0.618 |
|  | BMI | 0.126 | 0.155 | 0.003 | 0.302 | 0.031 | 0.008 | 0 | 0.372 | 0.031 | 0.01 | 0.031 | 0.008 | 0.012 | 0.376 |
|  | WC | 0.439 | 0.06 | 0.001 | 0.208 | 0.031 | 0.008 | 0.001 | 0.236 | 0.031 | 0.006 | 0.032 | 0.006 | 0.019 | 0.242 |
|  | HC | 0.251 | 0.189 | −0.001 | 0.565 | 0.031 | 0.008 | 0 | 0.572 | 0.032 | 4.00E−03 | 0.032 | 4.00E−03 | −0.006 | 0.572 |
|  | WHR | 0.002 | 0.157 | 0.447 | 0.005 | 0.031 | 0.008 | 0.001 | 0.12 | 0.03 | 2.00E−03 | 0.031 | 2.00E−03 | 0.033 | 0.122 |
| Total SCFAs | FI | 0.039 | 0.689 | 0.01 | 8.48E−05 | 0.032 | 3.70E−03 | 0 | 0.688 | 0.026 | 0.028 | 0.026 | 0.026 | 0.016 | 0.71 |
|  | HOMA-IR | 0.005 | 0.839 | 0.043 | 6.69E−05 | 0.032 | 3.70E−03 | 0 | 0.884 | 0.026 | 0.024 | 0.027 | 0.024 | 0.008 | 0.88 |
|  | HOMA-B | 1.486 | 0.252 | 0.001 | 2.24E−03 | 0.032 | 3.70E−03 | 0.001 | 0.246 | 0.026 | 0.032 | 0.027 | 0.024 | 0.034 | 0.258 |
|  | Hypertension | 0.006 | 0.565 | 0.085 | 1.39E−04 | 0.032 | 3.70E−03 | 0.001 | 0.548 | 0.031 | 0.01 | 0.032 | 0.01 | 0.017 | 0.55 |
|  | Dyslipidemia | 0.006 | 0.543 | 0.097 | 2.83E−05 | 0.032 | 3.70E−03 | 0.001 | 0.568 | 0.031 | 0.008 | 0.032 | 0.01 | 0.019 | 0.562 |
|  | TG | 0.025 | 0.237 | 0.074 | 5.85E−11 | 0.032 | 3.70E−03 | 0.002 | 0.214 | 0.03 | 2.00E−03 | 0.032 | 2.00E−03 | 0.059 | 0.216 |
|  | TC | 0.083 | 1.88E−03 | 0.021 | 0.022 | 0.032 | 3.70E−03 | 0.002 | 0.018 | 0.03 | 4.00E−03 | 0.032 | 0.00E+00 | 0.054 | 0.018 |
|  | LDL-C | 0.065 | 8.15E−04 | 0.021 | 0.094 | 0.032 | 3.70E−03 | 0.001 | 0.082 | 0.03 | 0.01 | 0.032 | 0.008 | 0.043 | 0.09 |
|  | HDL-C | 0.009 | 0.274 | −0.097 | 1.59E−03 | 0.032 | 3.70E−03 | −0.001 | 0.266 | 0.032 | 0.006 | 0.032 | 0.008 | −0.027 | 0.274 |
|  | Abdominal obesity | 0.01 | 0.353 | 0.06 | 0.009 | 0.032 | 3.70E−03 | 0.001 | 0.354 | 0.033 | 2.00E−03 | 0.033 | 0.00E+00 | 0.018 | 0.354 |
|  | Obesity | −0.009 | 0.285 | 0.068 | 0.024 | 0.032 | 3.70E−03 | −0.001 | 0.292 | 0.032 | 0.008 | 0.032 | 0.01 | −0.018 | 0.302 |
|  | BMI | −0.104 | 0.211 | 0.004 | 0.231 | 0.032 | 3.70E−03 | 0 | 0.326 | 0.032 | 0.008 | 0.032 | 0.01 | −0.011 | 0.336 |
|  | WC | −0.396 | 0.07 | 0.002 | 0.137 | 0.032 | 3.70E−03 | −0.001 | 0.2 | 0.032 | 0.006 | 0.032 | 0.006 | −0.021 | 0.206 |
|  | HC | −0.485 | 0.007 | 0 | 0.752 | 0.032 | 3.70E−03 | 0 | 0.712 | 0.033 | 2.00E−03 | 0.033 | 2.00E−03 | 0.006 | 0.714 |
|  | WHR | 0 | 0.908 | 0.459 | 4.32E−03 | 0.032 | 3.70E−03 | 0 | 0.958 | 0.033 | 0.006 | 0.033 | 0.006 | 0.002 | 0.964 |
| Propionate | FI | 0.196 | 0.046 | 0.01 | 1.26E−04 | 0.033 | 2.93E−03 | 0.002 | 0.032 | 0.029 | 0.012 | 0.031 | 0.008 | 0.065 | 0.04 |
|  | HOMA-IR | 0.045 | 0.062 | 0.042 | 1.02E−04 | 0.033 | 2.93E−03 | 0.002 | 0.078 | 0.029 | 0.01 | 0.031 | 4.00E−03 | 0.06 | 0.082 |
|  | HOMA-B | 2.919 | 0.026 | 0.001 | 2.85E−03 | 0.033 | 2.93E−03 | 0.002 | 0.018 | 0.03 | 0.008 | 0.031 | 4.00E−03 | 0.055 | 0.022 |
|  | Hypertension | 0.02 | 0.075 | 0.083 | 1.91E−04 | 0.033 | 2.93E−03 | 0.002 | 0.096 | 0.032 | 4.00E−03 | 0.033 | 4.00E−03 | 0.05 | 0.1 |
|  | Dyslipidemia | 0 | 0.985 | 0.097 | 2.39E−05 | 0.033 | 2.93E−03 | 0 | 0.984 | 0.033 | 4.00E−03 | 0.033 | 4.00E−03 | 0.001 | 0.984 |
|  | TG | 0.018 | 0.405 | 0.075 | 5.07E−11 | 0.033 | 2.93E−03 | 0.001 | 0.342 | 0.032 | 0.008 | 0.034 | 2.00E−03 | 0.04 | 0.344 |
|  | TC | 0.075 | 0.006 | 0.021 | 0.021 | 0.033 | 2.93E−03 | 0.002 | 0.018 | 0.032 | 0.008 | 0.033 | 4.00E−03 | 0.048 | 0.022 |
|  | LDL-C | 0.053 | 0.007 | 0.021 | 0.088 | 0.033 | 2.93E−03 | 0.001 | 0.082 | 0.032 | 0.012 | 0.034 | 0.012 | 0.034 | 0.094 |
|  | HDL-C | 0.01 | 0.224 | −0.098 | 1.52E−03 | 0.033 | 2.93E−03 | −0.001 | 0.212 | 0.035 | 0.008 | 0.034 | 0.012 | −0.029 | 0.224 |
|  | Abdominal obesity | −0.015 | 0.164 | 0.063 | 0.005 | 0.033 | 2.93E−03 | −0.001 | 0.166 | 0.035 | 2.00E−03 | 0.034 | 2.00E−03 | −0.029 | 0.168 |
|  | Obesity | −0.002 | 0.854 | 0.066 | 0.028 | 0.033 | 2.93E−03 | 0 | 0.866 | 0.033 | 4.00E−03 | 0.033 | 4.00E−03 | −0.003 | 0.862 |
|  | BMI | −0.043 | 0.615 | 0.003 | 0.25 | 0.033 | 2.93E−03 | 0 | 0.666 | 0.033 | 4.00E−03 | 0.033 | 4.00E−03 | −0.004 | 0.662 |
|  | WC | −0.384 | 0.085 | 0.002 | 0.137 | 0.033 | 2.93E−03 | −0.001 | 0.208 | 0.034 | 2.00E−03 | 0.034 | 2.00E−03 | −0.019 | 0.21 |
|  | HC | −0.246 | 0.179 | −0.001 | 0.685 | 0.033 | 2.93E−03 | 0 | 0.71 | 0.034 | 0.00E+00 | 0.034 | 0.00E+00 | 0.004 | 0.71 |
|  | WHR | −0.002 | 0.228 | 0.473 | 3.24E−03 | 0.033 | 2.93E−03 | −0.001 | 0.184 | 0.035 | 0.006 | 0.034 | 0.006 | −0.026 | 0.19 |
